# Adenocarcinoma Around the Esophagogastric Junction Mimicking Submucosal Tumors: A Case Report

**DOI:** 10.7759/cureus.91512

**Published:** 2025-09-02

**Authors:** Ryuki Ueda, Shoji Oura, Naoki Kataoka

**Affiliations:** 1 Department of Surgery, Kishiwada Tokushukai Hospital, Kishiwada, JPN

**Keywords:** ectopic gastric gland, gastric cancer, intact mucosae, internal high echoes, submucosal tumor

## Abstract

An 81-year-old man with a branch-type intraductal papillary neoplasm underwent endoscopic evaluation for dysphagia, leading to the assessment of esophageal candidiasis, esophageal stenosis around the esophagogastric junction, and no malignant findings on the esophageal and gastric mucosae. Computed tomography (CT) showed a submucosal tumor. Endoscopic ultrasound (EUS) of the presumed submucosal tumor showed a well-circumscribed mass with internal punctate high echoes and enhanced posterior echoes. Major parts of the mass showed yellow-green to red on endoscopic elastography. EUS-guided core needle biopsy pathologically showed atypical epithelial cells with cuboidal structures and stromal reaction, leading to the diagnosis of adenocarcinoma. The patient, therefore, underwent robotic-assisted proximal gastrectomy and lymph node dissection. Postoperative pathological study showed the positive nodes in the lower para-esophageal and right cardia lymph nodes and atypical cells growing mainly in a tubular fashion which lacked mucosal malignancy, penetrated beyond the muscular propria, and spread widely under the esophageal and gastric mucosae. Immunostaining was negative for HER-2, PD-L1, CLDN12, and MSI. The patient recovered uneventfully, was discharged on the 13th day after surgery, and is scheduled to be followed up on an outpatient basis without adjuvant chemotherapy due to his old age. Diagnostic physicians should note that gastric submucosal tumors with intact gastric mucosae and all with circumscribed margins, internal high echoes, and enhanced posterior echoes on EUS suggest the possible well-differentiated adenocarcinomas arising from ectopic gastric glands in the stomach.

## Introduction

Gastric submucosal tumors include benign diseases, such as lipomas and neurilemmomas, and malignant tumors, such as malignant lymphomas and sarcomas, with gastrointestinal stromal tumors (GISTs) being the most prevalent [1]. It, therefore, is very important for endoscopic specialists to properly diagnose and manage GISTs in the clinical practice of presumed gastric submucosal tumors. In diagnosing submucosal tumors, including GISTs, endoscopic biopsy is naturally very important for developing treatment strategies.

Diagnostic physicians can roughly diagnose epithelial tumors with endoscopic findings alone, but are limited to evaluating their location and size in submucosal tumors. Endoscopic ultrasound (EUS), therefore, is very useful for diagnostic physicians to get much more diagnostic information about the submucosal tumors [2]. Unlike ultrasound specialists, however, endoscopic specialists often have less knowledge about the correlation between ultrasound findings and pathological characteristics of submucosal tumors.

Ultrasound can not only judge the location, size, and margin clarity of masses but also speculate the pathological components of the mass by evaluating the internal echoes, posterior echoes, and margin status. In short, internal punctate high echoes highly suggest the microvoid-containing pathological components such as tubular or papillary structures [3]. Conversely, internal low echoes, attenuated posterior echoes, and obscured tumor margins suggest the intra-tumoral presence of fibrous components [4].

We herein report an extremely rare gastric well-differentiated adenocarcinoma case judged to have originated from the ectopic gastric gland in the stomach with EUS.

## Case presentation

**Figure 1:**
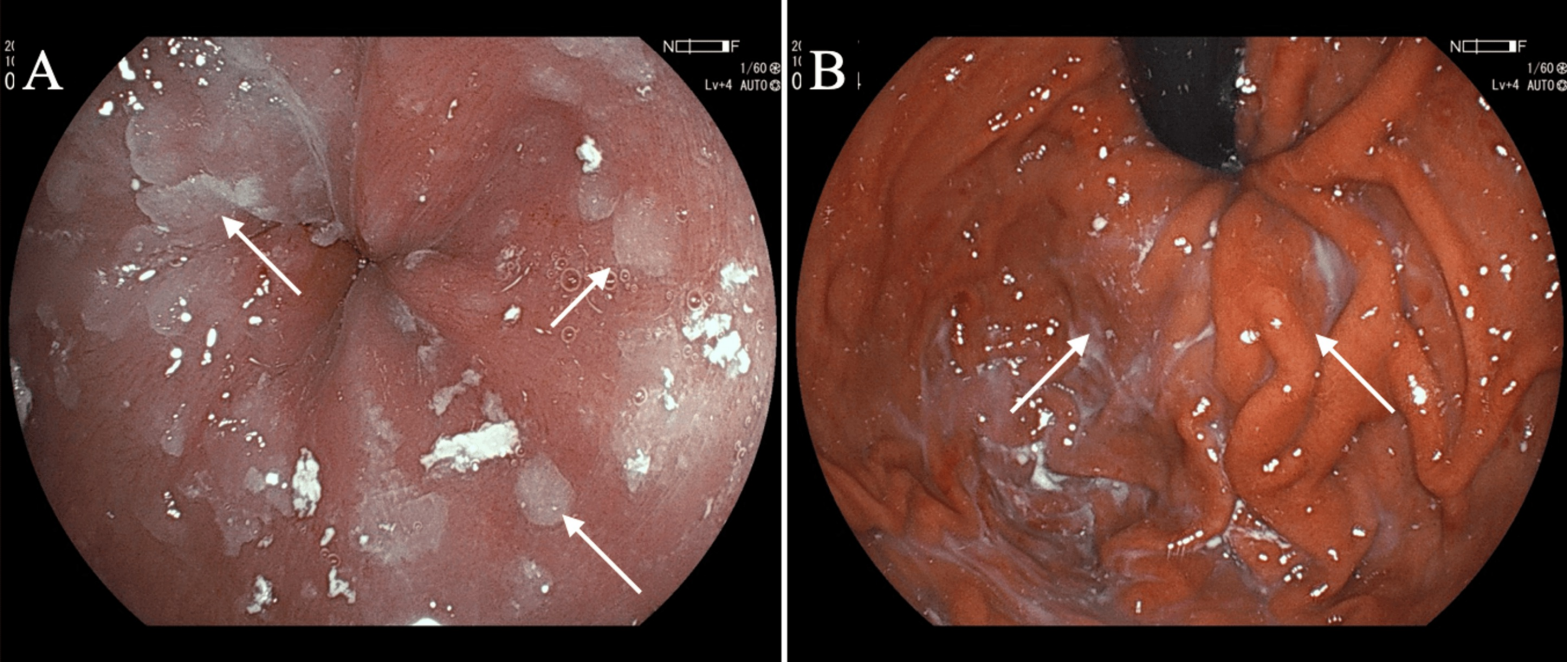
Endoscopic findings (A) Many presumed candida-induced white plaques (arrows) were observed on the esophageal mucosae. No tumorous lesions, however, were detected on the esophageal mucosae. (B) Upper gastrointestinal tract endoscopy showed no mucosal abnormalities but a presumed submucosal tumor (arrows) around the esophagogastric junction.

An 81-year-old man with a branch-type intraductal papillary neoplasm judged by EUS had been followed up with various diagnostic images. Dysphagia lasting two months made the patient undergo endoscopic evaluation, leading to the assessment of esophageal candidiasis, esophageal stenosis around the esophagogastric junction, and no malignant findings of the esophageal and gastric mucosae (Figure 1).

**Figure 2:**
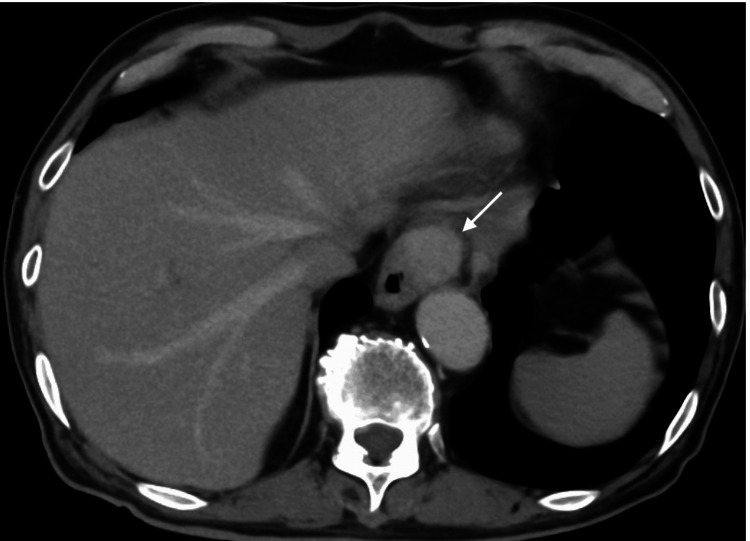
CT findings CT showed an enhanced mass (arrow) at the cardia. CT: computed tomography

Computed tomography (CT) showed a presumed submucosal tumor (Figure 2).

**Figure 3:**
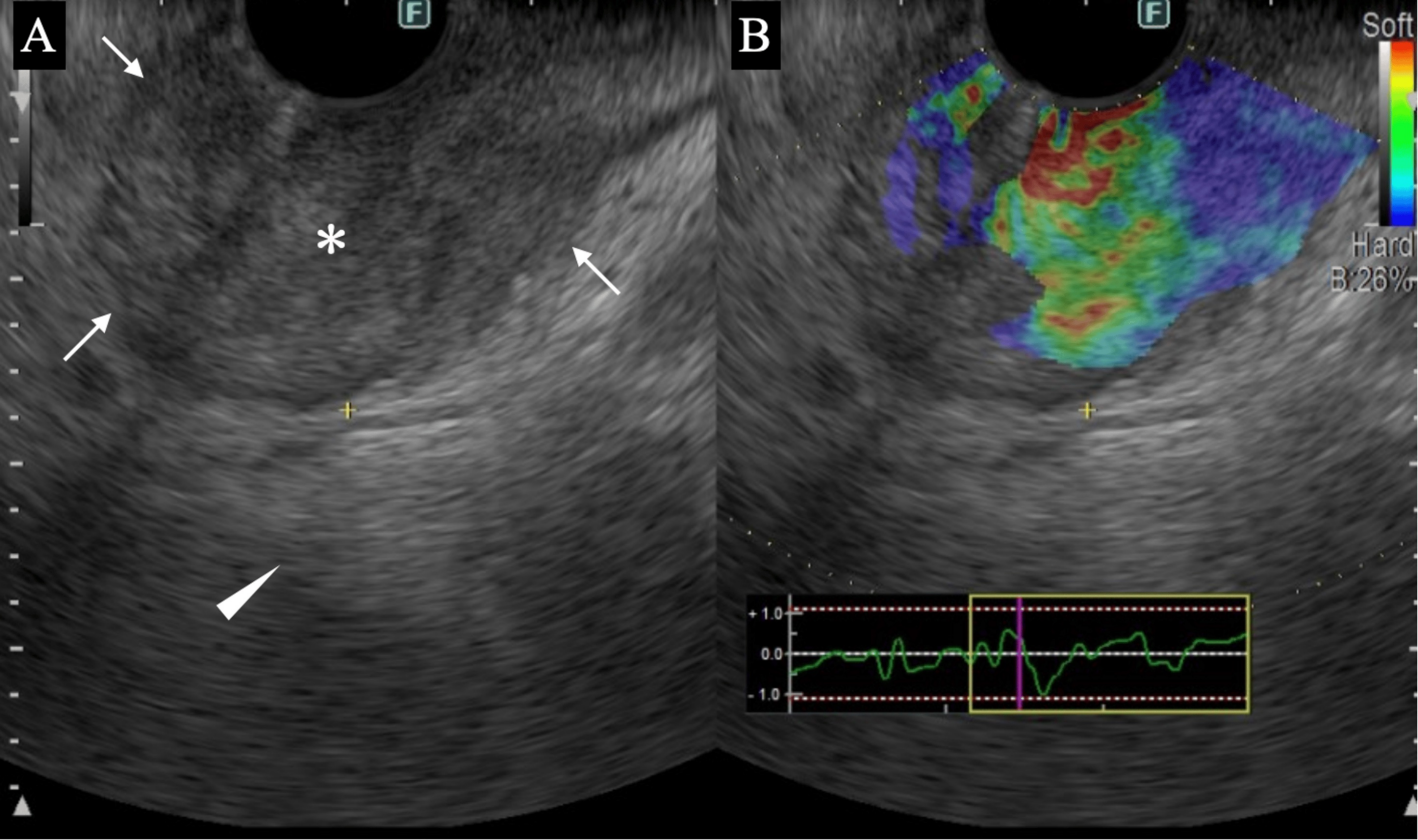
EUS findings (A) EUS showed a circumscribed oval mass (arrows) with internal high echoes (asterisk) and enhanced posterior echoes (arrowhead). (B) Elastography showed no blue parts at least in the mass center. EUS: endoscopic ultrasonography

EUS showed a well-circumscribed mass with internal punctate high echoes and enhanced posterior echoes (Figure 3).

Major parts of the mass showed yellow-green to red on endoscopic elastography. EUS-guided core needle biopsy pathologically showed atypical epithelial cells with cuboidal structures and stromal reaction and proved the submucosal tumor to be an adenocarcinoma. Laboratory tests showed a normal carcinoembryonic antigen (CEA) level of 2.7 ng/mL (reference range: 0-5 ng/mL) and an elevated CA19-9 level of 289 U/mL (reference range: 0-37 U/mL) (Table 1).

**Table 1:** Blood test AST: aspartate aminotransferase; ALT: alanine aminotransferase; LDH: lactate dehydrogenase; ALP: alkaline phosphatase; γ-GTP: γ-glutamyl transpeptidase; Ch-E: cholinesterase; CK: creatine kinase; CRP: C-reactive protein; WBC: white blood cell count; RBC: red blood cell count; Hb: hemoglobin; Ht: hematocrit; CEA: carcinoembryonic antigen; AFP: alpha-fetoprotein

Test	Reference range	Result
Total bilirubin (ng/dL)	0.4-1.5	0.9
AST (U/L)	13-30	21
ALT (U/L)	7-23	17
LDH (U/L)	124-222	155
ALP (U/L)	38-113	53
γ-GTP (U/L)	9-32	12
Ch-E (U/L)	201-421	261
CK (U/L)	41-153	58
Amylase (U/L)	33-132	99
Total protein (g/dL)	6.6-8.1	6.3
Albumin (U/DL)	4.1-5.1	3.7
CRP (mg/dL)	0-0.14	0.05
WBC (10×2/μL)	33-86	40
RBC (10×4/μL)	386-492	478
Hb (g/dL)	11.6-14.8	14.6
Ht (%)	35.1-44.4	43.8
Platelet (10×4/μL)	15.8-34.8	21
CEA (ng/mL)	0-5	2.7
AFP (ng/mL)	0-7.9	2.3
CA19-9 (U/mL)	0-37	289

**Figure 4:**
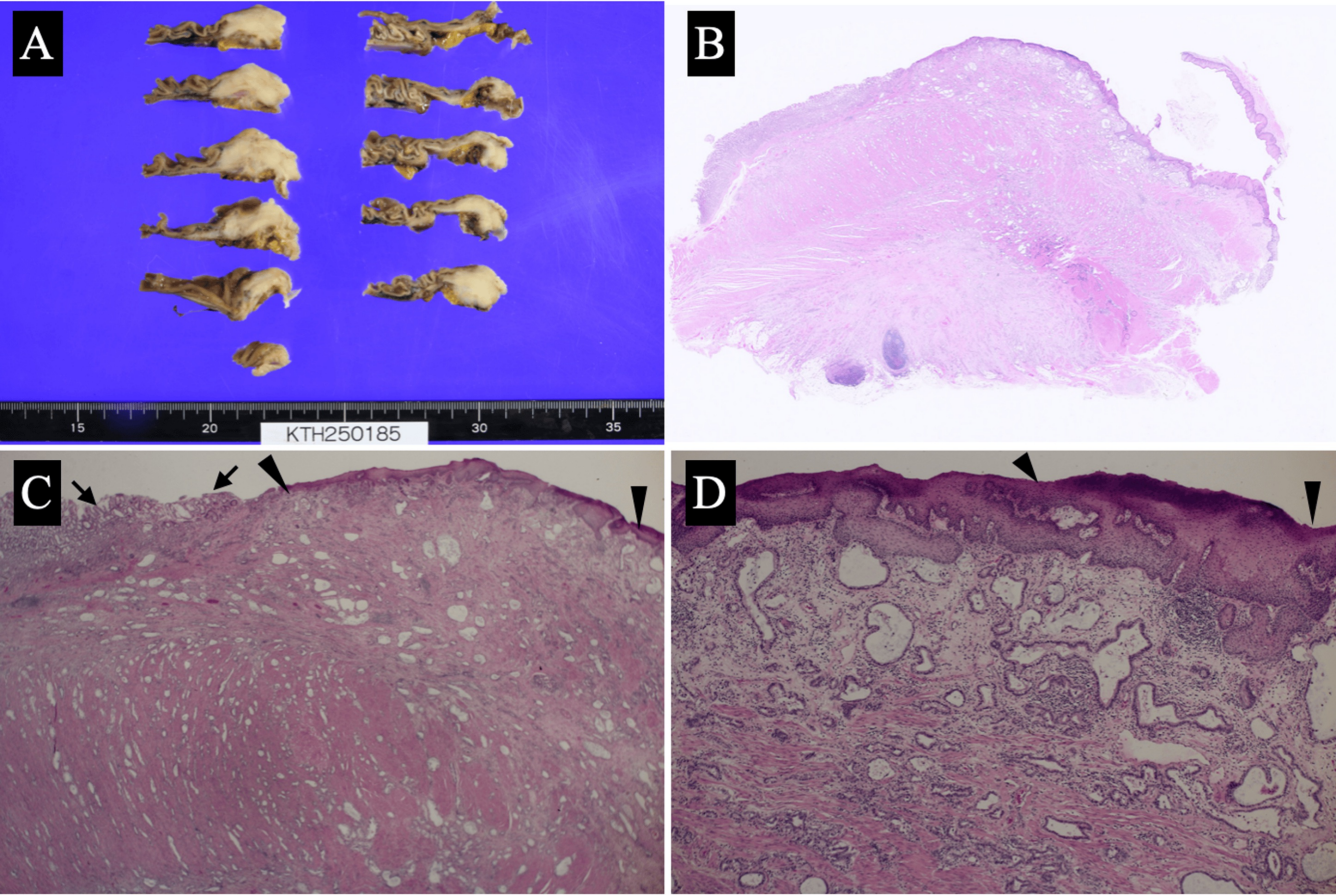
Histological findings (A) The tumor was located beneath the esophageal and gastric mucosae. (B) Low magnified view showed that the tumor was located under the esophagogastric mucosae. (C) The tumor had atypical cells mainly growing in a tubular fashion, which spread widely in the submucosae and had atypical cells in neither the gastric (arrows) nor esophageal (arrowheads) mucosae. (D) Magnified view showed atypical epithelial cells growing in a tubular fashion and no epithelial malignancies on both the gastric and esophageal (arrowheads) mucosae.

The patient, therefore, underwent robotic-assisted proximal gastrectomy and lymph node dissection followed by gastrointestinal reconstruction using the modified SOFY technique [5] under the tentative diagnosis of adenocarcinoma around the esophagogastric junction despite the lack of mucosal malignancy in both the esophagus and stomach. Postoperative pathological study showed three positive nodes in the lower para-esophageal and right cardia lymph nodes and atypical cells growing mainly in a tubular fashion which lacked mucosal malignancy, penetrated beyond the muscular propria, and spread widely under the esophageal and gastric mucosae (Figure 4).

Immunostaining was negative for HER-2, PD-L1, CLDN12, and MSI. The patient recovered uneventfully, was discharged on the 13th day after surgery, had a normalization of the elevated CA19-9 level, and is scheduled to be followed up on an outpatient basis without adjuvant chemotherapy due to his old age.

## Discussion

Combination assessment of shapes, internal echoes, and posterior echoes of masses can give diagnostic physicians important information about the pathological characteristics of the mass [6,7]. In short, reflection, backscattering, and attenuation of ultrasound waves determine shapes, internal echoes, and posterior echoes of masses, respectively. These three ultrasound findings, therefore, can make us speculate on the pathological components in the mass. Elasticity of the mass can give diagnostic physicians further suggestions about the pathological characteristics of the mass. It is well known that fibrous components at the mass borders obscure the tumor margins [8]. Scirrhous-type invasive ductal breast carcinomas, therefore, generally have speculated shapes. EUS clearly showed that the mass had distinct margins in this case, highly suggesting the lack or least presence of fibrous components at the mass borders. In other words, the tumor in this case can be speculated as a cell-rich mass.

EUS showed enhanced posterior echoes of the mass. Various pathological components can attenuate ultrasound waves and cause posterior echo attenuation. Of the many pathological components present in masses, fibrous components can most efficiently attenuate the ultrasound waves [8,9]. Conversely, the lack of fibrous components can generally make enhanced posterior echoes. It was evident that the present tumor had no or very sparse, if present, fibrous components both at the tumor margins and in the tumor.

Internal echoes of the tumor were mainly comprised of numerous punctate high echoes. Internal high echoes are generally caused by the presence both of pathological components with different acoustic impedance from those of tumor cells, e.g., fat cells, and of microvoids comprising papillary or tubular structures [10]. The tumor actually had atypical cells growing mainly in a tubular fashion, which spread diffusely in the mass. Conversely, GISTs generally have spindle cells with similar acoustic impedance growing in an expansive manner, which cause least ultrasound wave backscattering, generate internal low echoes, and extremely rarely develop internal high echoes [11].

Elastography showed no parts expressed as blue in the tumor, which indicated no or sparse presence of fibrous components in the tumor [12]. We, therefore, can judge that this submucosal tumor had scarce fibrous components in it based on all the ultrasound findings of margin clarity, internal high echoes, enhanced posterior echoes, and mass softness.

Despite the lack of epithelial malignancy on both the esophageal and gastric mucosae, this tumor had well-differentiated adenocarcinoma cells widely spreading submucosally around the esophagogastric junction. These findings suggest two possible mechanisms [13]. The first mechanism is that adenocarcinoma cells that developed in the gastric mucosa submucosally spread widely and then epithelial cancer cells disappear.** **The second mechanism is that cancer cells originate from the ectopic gastric glands in the stomach and then spread widely and submucosally. The former mechanism generally develops poorly differentiated adenocarcinomas or signet ring cell carcinomas, which markedly differed from the pathological findings observed in this case. Adenocarcinoma cells caused by the latter mechanism are reported to often develop large cystic lesions in the gastric walls in many studies [11-13] but can have no cystic lesions. The presence of well-differentiated papillary lesions easily makes us imagine that gastric juice secretion from ectopic gastric glands is inhibited, forming cyst-like lesions. The small tubular adenocarcinoma structures suggest low gastric juice production, which might have led to the non-formation of cystic lesions in the gastric wall in this case.

Pathological examination of endoscopic biopsy tissue is naturally very important for definitive diagnosis in submucosal tumors. However, by predicting the pathological components based on ultrasound findings, diagnostic physicians can correctly diagnose rare disorders like this case. It, therefore, is imperative for endoscopists to be familiar with the correlation between ultrasound and pathological findings.

## Conclusions

Some gastric cancers arising from ectopic gastric glands in the stomach can have no cystic structures in the gastric walls. In addition, circumscribed tumor margins, internal high echoes, and enhanced posterior echoes on EUS suggest the possible well-differentiated adenocarcinoma arising from ectopic gastric glands in the stomach. Endoscopic specialists, therefore, should be familiar not only with endoscopic findings but also with the interpretation of EUS findings.
